# Complete Mitochondrial Genome Sequence of *Aethina tumida* (Coleoptera: Nitidulidae), a Beekeeping Pest

**DOI:** 10.1128/genomeA.01165-17

**Published:** 2017-11-02

**Authors:** Véronique Duquesne, Aurélie Delcont, Anthéa Huleux, Véronique Beven, Fabrice Touzain, Magali Ribière-Chabert

**Affiliations:** a Anses, Laboratory of Sophia-Antipolis, European Reference Laboratory for Honey Bee Health, Honey Bee Pathology Unit, Sophia-Antipolis, France; b Anses, Laboratory of Ploufragan, Unit of Viral Genetics and Biosafety, Ploufragan, France

## Abstract

We report here the full mitochondrial genome sequence of *Aethina tumida*, a Nitidulidae species beetle, that is a pest of bee hives. The obtained sequence is 16,576 bp in length and contains 13 protein-coding genes, 2 rRNA genes, and 22 tRNAs.

## GENOME ANNOUNCEMENT

The small hive beetle (SHB), *Aethina tumida* Murray 1867 (Coleoptera: Nitidulidae), is an invasive pest of bee hives originally from sub-Saharan Africa. It was introduced in many different countries (1, 2) throughout the world, which led to severe damage to honeybee colonies, especially in North America. The first introduction of this beetle to a European country occurred in Italy in 2014 (3). The mitochondrial DNA (mtDNA) is widely used in phylogenetic and phylogeographic studies. In the case of *A. tumida*, the sequencing of the cytochrome oxydase I (COI) gene allowed the identification of the origin of the specimen introduced into Italy (4). The first data on the *A. tumida* complete genome were obtained recently using a transcriptomic analysis (5). However, no data regarding the organization of the mitochondrial DNA are available to date. This seems essential to complete genetic information about this beetle to determine its genetic diversity and manage its introduction.

The specimen of *A. tumida* used for this study was collected from South Africa and confirmed by the Biosystematics Division of the Plant Protection Research Institute (Pretoria, South Africa). The sequencing strategy used corresponded to a mix of three technologies. The genomic DNA was first obtained from a single whole specimen and used for library preparation. Reads were obtained on an Ion Proton sequencer, were cleaned with Trimmomatic 0.32 (6), and were aligned against available insect mitochondrial sequences using tblastn. Matching reads were *de novo* assembled with SPAdes 3.1.1 (7) to obtain 20 contigs.

Then, to complete the sequence, different primers were designed and used for bridging the gaps. The PCR fragments were sequenced by Sanger sequencing. A total of 12,988 bp was annotated. However, a large section containing the two rRNA genes and the control region were still missing. Consequently, a new DNA preparation was performed with the aim of avoiding bacterial contamination by removing the entire abdominal segment. A new library was produced with Hybrigenics-Helixio (Clermont-Ferrand, France) using the Nextera XT DNA Library kit (Illumina), and paired-end sequencing was performed on a NextSeq500 (Illumina). The obtained reads were aligned with the 12,988-bp sequence as a reference and the putative mitochondrial contigs from the SHB transcriptome (5). A novel *de novo* assembly allowed the obtention of a 16,576-bp complete sequence. Annotation was done using MITOS (8), manually verified by BLAST analysis against GenBank, and compared to the available mitochondrial genome of a Nitidulidae sp. (GenBank accession number KT696260) as the closest sequence.

The mtDNA genome of *A. tumida* is a typical circular molecule of 16,576 bp in length containing genes for the 13 conserved mitochondrial proteins for oxidative phosphorylation (*atp6*, *atp8*, *cytb*, *cox1*, *cox2*, *cox3*, *nad1*, *nad2*, *nad3*, *nad4*, *nad4L*, *nad5*, and *nad6*), 2 rRNA genes, and 22 tRNA genes. In addition, a large noncoding region (1,906 bp) is located between *rrnS* and *trnI-trnQ-trnM*. The CG content is 23.1%. This full mitochondrial genome organization is important for understanding propagation and developing new tools for diagnosis and pest control.

### Accession number(s).

The SHB mtDNA genome has been deposited in GenBank under accession number MF943248.

## References

[1] Sanford MT 1998 *Aethina tumida*: a new beehive pest in the Western Hemisphere. Apis 16:1–5.

[2] Neumann P, Ellis JD 2008 The small hive beetle (*Aethina tumida* Murray, Coleoptera: Nitidulidae): distribution, biology and control of an invasive species. J Apic Res 47:181–183. doi:10.1080/00218839.2008.11101453.

[3] Mutinelli F, Montarsi F, Federico G, Granato A, Ponti AM, Grandinetti G, Ferrè N, Franco S, Duquesne V, Rivière M, Thiéry R, Henrikx P, Ribière-Chabert M, Chauzat M 2014 Detection of *Aethina tumida* Murray (Coleoptera: Nitidulidae) in Italy: outbreaks and early reaction measures. J Apic Res 53:569–575. doi:10.3896/IBRA.1.53.5.13.

[4] Granato A, Zecchin B, Baratto C, Duquesne V, Negrisolo E, Chauzat MP, Ribière-Chabert M, Cattoli G, Mutinelli F 2017 Introduction of *Aethina tumida* (Coleoptera: Nitidulidae) in the regions of Calabria and Sicily (southern Italy). Apidologie 48:194–203. doi:10.1007/s13592-016-0465-3.

[5] Tarver MR, Huang Q, de Guzman L, Rinderer T, Holloway B, Reese J, Weaver D, Evans JD 2016 Transciptomic and functional resources for the small hive beetle *Aethina tumida*, a worldwide parasite of honey bees. Genomics Data 9:97–99. doi:10.1016/j.gdata.2016.06.003. 27453819 PMC4941039

[6] Bolger AM, Lohse M, Usadel B 2014 Trimmomatic: a flexible trimmer for Illumina sequence data. Bioinformatics 30:2114–2120. doi:10.1093/bioinformatics/btu170. 24695404 PMC4103590

[7] Bankevich A, Nurk S, Antipov D, Gurevich AA, Dvorkin M, Kulikov AS, Lesin VM, Nikolenko SI, Pham S, Prjibelski AD, Pyshkin AV, Sirotkin AV, Vyahhi N, Tesler G, Alekseyev MA, Pevzner PA 2012 SPAdes: a new genome assembly algorithm and its applications to single-cell sequencing. J Comput Biol 19:455–477. doi:10.1089/cmb.2012.0021. 22506599 PMC3342519

[8] Bernt M, Donath A, Jühling F, Externbrink F, Florentz C, Fritzsch G, Pütz J, Middendorf M, Stadler PF 2013 MITOS: improved *de novo* Metazoan mitochondrial genome annotation. Mol Phylogenet Evol 69:313–319. doi:10.1016/j.ympev.2012.08.023. 22982435

